# The NASSS (Non-Adoption, Abandonment, Scale-Up, Spread and Sustainability) framework use over time: A scoping review

**DOI:** 10.1371/journal.pdig.0000418

**Published:** 2025-03-17

**Authors:** Hwayeon Danielle Shin, Emily Hamovitch, Evgenia Gatov, Madison MacKinnon, Luma Samawi, Rhonda Boateng, Kevin E. Thorpe, Melanie Barwick

**Affiliations:** 1 Institute of Health Policy, Management, and Evaluation, University of Toronto, Toronto, Ontario, Canada; 2 Krembil Centre for Neuroinformatics, Centre for Addiction and Mental Health, Toronto, Ontario, Canada; 3 The Centre for Addiction and Mental Health, Toronto, Ontario, Canada; 4 Child Health Evaluative Sciences, The Peter Gilgan Centre for Research and Learning, The Hospital for Sick Children, Toronto, Ontario, Canada; 5 Department of Psychiatry, University of Toronto, Toronto, Ontario, Canada; Massachusetts General Hospital, COLOMBIA

## Abstract

The Non-adoption, Abandonment, Scale-up, Spread, Sustainability (NASSS) framework (2017) was established as an evidence-based, theory-informed tool to predict and evaluate the success of implementing health and care technologies. While the NASSS is gaining popularity, its use has not been systematically described. Literature reviews on the applications of popular implementation frameworks, such as the RE-AIM and the CFIR, have enabled their advancement in implementation science. Similarly, we sought to advance the science of implementation and application of theories, models, and frameworks (TMFs) in research by exploring the application of the NASSS in the five years since its inception. We aim to understand the characteristics of studies that used the NASSS, how it was used, and the lessons learned from its application. We conducted a scoping review following the JBI methodology. On December 20, 2022, we searched the following databases: Ovid MEDLINE, EMBASE, PsychINFO, CINAHL, Scopus, Web of Science, and LISTA. We used typologies and frameworks to characterize evidence to address our aim. This review included 57 studies that were qualitative (n=28), mixed/multi-methods (n=13), case studies (n=6), observational (n=3), experimental (n=3), and other designs (e.g., quality improvement) (n=4). The four most common types of digital applications being implemented were telemedicine/virtual care (n=24), personal health devices (n=10), digital interventions such as internet Cognitive Behavioural Therapies (n=10), and knowledge generation applications (n=9). Studies used the NASSS to inform study design (n=9), data collection (n=35), analysis (n=41), data presentation (n=33), and interpretation (n=39). Most studies applied the NASSS retrospectively to implementation (n=33). The remainder applied the NASSS prospectively (n=15) or concurrently (n=8) with implementation. We also collated reported barriers and enablers to implementation. We found the most reported barriers fell within the Organization and Adopter System domains, and the most frequently reported enablers fell within the Value Proposition domain. Eighteen studies highlighted the NASSS as a valuable and practical resource, particularly for unravelling complexities, comprehending implementation context, understanding contextual relevance in implementing health technology, and recognizing its adaptable nature to cater to researchers’ requirements. Most studies used the NASSS retrospectively, which may be attributed to the framework’s novelty. However, this finding highlights the need for prospective and concurrent application of the NASSS within the implementation process. In addition, almost all included studies reported multiple domains as barriers and enablers to implementation, indicating that implementation is a highly complex process that requires careful preparation to ensure implementation success. Finally, we identified a need for better reporting when using the NASSS in implementation research to contribute to the collective knowledge in the field.

## Author summary

In this review, we explored how the Non-adoption, Abandonment, Scale-up, Spread, and Sustainability (NASSS) framework has been used in health and care technology research since its introduction in 2017. The NASSS helps researchers and practitioners understand why some healthcare innovations succeed while others fail. We reviewed 57 studies to see how the framework was applied, what types of technologies it supported, and what lessons were learned from its use. Most studies used the NASSS after implementation to reflect on what worked and what did not, suggesting that researchers are still learning how to apply it during the planning stages. We found that common barriers to successful implementation often occurred within organizations and among the people expected to adopt new technologies, while success was more likely when the technology was perceived as valuable (for example, through benefits such as improved patient outcomes, increased access to care, or enhanced organizational processes). Our findings highlight the importance of using frameworks like the NASSS earlier in the process to anticipate challenges and improve outcomes. We also emphasize the need for clearer reporting when using the NASSS so that others can learn from these experiences and continue to improve how healthcare technologies are implemented in real-world settings.

## Introduction

Healthcare technology innovations hold considerable promise for enhancing patient outcomes and service efficiency, but they frequently remain confined to small-scale demonstration initiatives [1–5]. Moreover, current evidence indicates a prevalent pattern of non-adoption and abandonment of healthcare technology innovations by their intended users, with limited success in integrating these innovations into regular practice or expanding their implementation to different contexts [6]. This challenge is especially evident in complex healthcare settings, where the multifaceted nature of the innovations and the environment can create barriers to successful implementation [7].

Healthcare is described as a complex adaptive system, discouraging simplistic linear cause-and-effect reasoning [8,9]. Instead, there is a growing recognition of the need to emphasize dynamic processes while implementing healthcare practices. This change in perspective reflects an understanding that healthcare is influenced by multifaceted interactions and feedback loops that cannot be adequately explained by linear models alone. In response to this evolving perspective, the Non-Adoption, Abandonment, Scale-up, Spread, and Sustainability (NASSS) framework was introduced in 2017 [10]. The NASSS was developed as an evidence-based and theory-informed approach to enhance the ability to predict and assess the success of implementing innovative technologies in healthcare [10]. Related complexity assessment tools (i.e., NASSS-CAT) were developed in 2020 to enhance understanding, guide monitoring, and facilitate research on technology projects in healthcare or social care settings through collaborations [11].

The NASSS encompasses seven distinct domains: 1) Illness/Condition; 2) Technology; 3) Value Proposition; 4) Adopter System; 5) Organization(s); 6) Wider Context; and 7) Embedding and Adaptation Over Time [10]. Each domain can be categorized as simple, complicated, or complex [10]. The greater the complexity observed within these domains, the more obstacles will likely arise, hindering the successful adoption, scale-up, spread, and sustainability of innovative health and care technologies [10]. The NASSS framework considers the intricate web of dynamic interactions that influence the adoption and outcomes of innovations and aims to provide a more comprehensive and accessible tool for evaluating and improving the implementation of healthcare innovations [10].

Although new, the NASSS framework has been well-received. As reported in the *Journal of Medical Internet Research* [10]*,* the seminal paper has had nearly 1060 citations at the time of writing. The surge in interest reflects the widespread adoption of the NASSS, which has been utilized prospectively and retrospectively to assess patient-oriented technologies and tools for decision-making purposes [12,13]. For example, Gremyr et al. [12] applied the NASSS and identified various implementation complexities across several domains for their point-of-care dashboard supporting schizophrenia care. Then, they used the NASSS-CAT to generate recommendations for the development and deployment of the dashboard [12]. Their analysis revealed the need for a clear value proposition that includes detailed information on costs, benefits, and risks. This can help guide decisions on allocating additional resources or discontinuing further development [12]. As such, NASSS’s utility has gained popularity in implementation research. However, there has been a lack of systematic documentation regarding the use of the NASSS framework following its release. Likewise, a comprehensive analysis of the framework’s contributions and the insights derived from its application has not been conducted systematically.

The applications of popular implementation theories, models, and frameworks (TMFs), such as the Reach, Effectiveness, Adoption, Implementation, and Maintenance (RE-AIM) and the Consolidated Framework for Implementation Research (CFIR), have been well documented in the literature. For example, several literature reviews have been written on the RE-AIM since its inception in 1999 [14,15]. These reviews have described and assessed the application of the RE-AIM, enabling its advancement (e.g., the enhanced RE-AIM/Pragmatic Robust Implementation and Sustainability Model (PRISM) 2019) and novel applications, such as combining the RE-AIM with the Pragmatic Explanatory Continuum Indicator Summary (PRECIS) model [14,15]. Similarly, we aimed to contribute to the field of implementation science by exploring the NASSS framework’s applications to date and identifying opportunities to advance the framework. A scoping review methodology was deemed most appropriate because our primary objective was to provide a breadth of literature currently available on the NASSS application [16]. A preliminary search of PROSPERO, MEDLINE, the Cochrane Database of Systematic Reviews, Open Science Framework, and *JBI Evidence Synthesis* was conducted in October 2022. No current or in-progress scoping or systematic reviews on the topic were identified.

### Review questions

1. What are the characteristics of studies that used the NASSS framework?
2. How has the NASSS framework been used in the identified studies, including, but not limited to, timing within implementation, depth of application, and use in combination with other tools (e.g., the NASSS-CAT)?
3. What are the author-reported lessons learned from applying the NASSS framework?

### Inclusion and exclusion criteria

#### Concept.

This review included all studies that used the NASSS and/or NASSS-CAT framework to inform the overall research design, data collection, analysis, presentation or interpretation. Studies that referred to the NASSS framework but did not apply it were excluded. This included instances where the framework was mentioned only in the introduction or discussion sections of the paper rather than being actively used as a methodological or analytical tool.

#### Context and population.

There were no exclusion criteria for population and context. Any studies conducted in any context with any population were considered for inclusion. However, due to the available resources in our research team, only English-language publications were included.

#### Type of sources.

This review included all research designs (e.g., quantitative, observational, qualitative, and mixed methods). We also considered peer-reviewed and grey literature, including conference proceedings and dissertations, but we included only empirical studies. Non-empirical literature, such as commentaries, conceptual papers, books, and literature reviews, was excluded. Reference lists in non-empirical literature (e.g., reviews) were screened to identify relevant primary empirical studies. Only literature published since 2017, the year of the publication of the seminal NASSS framework paper, was included.

## Methods

This scoping review was conducted following the JBI methodology for scoping reviews [17,18], and the manuscript was prepared in line with the Preferred Reporting Items for Systematic Reviews and Meta-Analyses extension for Scoping Reviews (PRISMA-ScR) [19]. Our *a priori* protocol [20] was registered on the Open Science Framework.

### Search strategy

In collaboration with a health sciences librarian and following the Peer Review of Electronic Search Strategies (PRESS) guideline [21], a comprehensive search strategy was developed to locate relevant scholarly literature using multiple bibliographic databases. The following describes how our search strategy was refined and how it underwent iterative steps before being validated by the librarian using the PRESS guideline. This scoping review followed a three-step search strategy outlined in the JBI methodology. Firstly, an initial limited search of MEDLINE was undertaken to identify articles on the topic. Secondly, the text words in the titles and abstracts of relevant articles and the index terms used to describe the articles were used to develop a complete search strategy. The search string included terms related to the NASSS framework (“NASSS,” “NASSS-CAT”). We looked for articles containing the following words (i.e., non-adoption, abandonment, scale-up, spread, sustainability, Greenhalgh, framework, model) in their titles, abstracts, or keywords. Additionally, we used proximity operators (e.g., (Greenhalgh* NEAR/5 (framework* OR model*)). Then, the entire search strategy, including all identified keywords and index terms, was adapted according to each database. Our search was undertaken on December 20, 2022, in the following databases: Ovid MEDLINE, EMBASE, PsychINFO, CINAHL, Scopus, Web of Science, and Library, Information Science and Technology Abstracts (LISTA). Thirdly, reference lists of relevant reviews were screened to identify eligible empirical studies. The complete search strategies are provided in S1 Appendix. Since the NASSS framework was first published in 2017, databases have been searched from 2017 onwards. In addition to a scholarly database search, a forward citation search [22] was used in Scopus and Web of Science on October 13 and 17, 2022, to complement our database searches. The main steps in this forward citation search included using citation indexes to identify studies that cite the original NASSS framework paper published in 2017. This search strategy helped identify papers our database searches might have missed.

### Study/source of evidence selection

All identified records were collated and uploaded into the Covidence [23], and duplicates were automatically removed. Then, five random articles were selected for our pilot testing. All six reviewers on the team independently assessed the titles and abstracts against the inclusion criteria. After pilot testing for the calibration exercise, the remaining titles and abstracts were each screened by two independent reviewers (HDS, EG, MM, EH, LS, RB). Relevant papers were retrieved in full, and their citation details were imported into the Covidence [23]. Two independent reviewers assessed full texts (HDS, EG, MM, EH, LS, RB). Full-text studies that did not meet the inclusion criteria were excluded, and reasons for their exclusion were documented. Any reviewer disagreements were resolved through discussion or with a third reviewer. Scoping reviews typically do not necessitate methodological evaluation [18]; therefore, critical appraisal was omitted.

### Data extraction

Teams of two independent reviewers (HDS, EH, EG, MM, RB, LS) extracted data using a data extraction tool developed in collaboration with the research team. We extracted the general characteristics of the paper, intervention characteristics, a description of the NASSS framework application, reported implementation barriers and enablers, study conclusions, and author-reported lessons learned from applying the framework. Any reviewer disagreements were resolved through discussion or with a third reviewer. See S2 Appendix for our data extraction tool.

### Data analysis and presentation

A descriptive, analytical approach was used to generate summary statistics (e.g., frequency counts, percentages, etc.) using Microsoft Excel for the general characteristics of the included studies. Subsequently, a content analysis was conducted to characterize the narrative data using the Excel Spreadsheet. First, the digital applications implemented in the included studies were categorized by two reviewers (MM, HDS) by adapting the framework, ‘Evolving Applications of Digital Technology in Health and Health Care’ [24]. Application categories [24] are as follows: 1) Virtual care; 2) Personal health devices; 3) Digital interventions; 4) Knowledge generation and/or integrators; 5) Health information; 6) Surgical/Radio graphic interventions; 7) Diagnostic and imaging [24]. Each innovation could be mapped onto more than one category. Secondly, two reviewers (EH, HDS) categorized health conditions examined in the included studies into disease types. Thirdly, the description of the NASSS framework application in each article was assessed by teams of two independent reviewers (HDS, EH, EG, MM, RB, LS) in terms of its timing within the implementation (i.e., prospective, retrospective, concurrent) and study design aspects (e.g., overall design, data collection, data analysis). This process required some level of interpretation by the team, and any conflicts in interpretation were resolved through discussion with the remaining team members. Fourth, barriers and enablers, often correspondingly reported to the primary NASSS domains, were collated. Then, teams of two reviewers (HDS, EH, EG, MM, RB) categorized these into subdomains of the NASSS framework. Fifth, reported lessons learned from the authors were narratively summarized. The charted results are accompanied by narrative summaries that describe how the results relate to our review objectives and questions.

## Results

**Fig 1:**
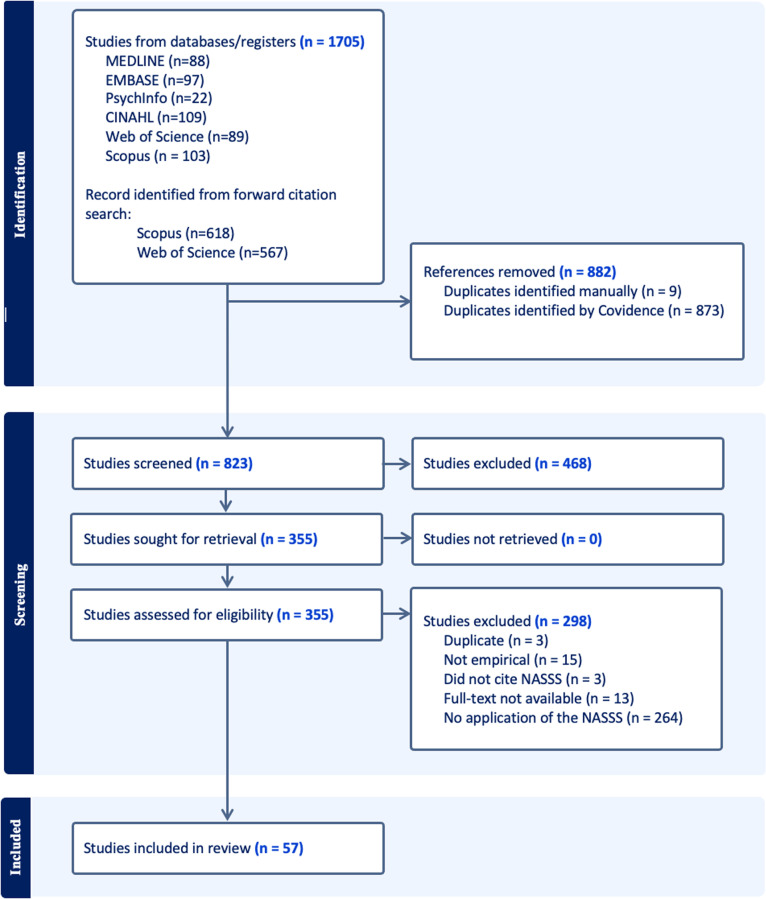
PRISMA flow diagram.

Our search strategy yielded 1,705 citations (Fig 1). Following the automatic removal of duplicates by Covidence, 823 articles underwent title and abstract screening. Then, 355 articles underwent full-text evaluation, culminating in 57 studies in this review. Most excluded studies cited the NASSS framework in the text (e.g., in the discussion) but did not use the framework to inform the study design, data collection, analyses, presentation, or interpretation. Other excluded studies were non-empirical (e.g., commentary), or their full text was unavailable.

### RQ1. Characteristics of included studies

Individual study characteristics are presented in Table 1. As indicated in summary Table 2, among the 57 included studies, the majority were qualitative (n=28), following mixed/multi-methods (n=13), case-studies (n=6), observational (n=3), experimental (n=3), and other designs (e.g., quality improvement, n=4). Many studies originated in the United Kingdom (n=15), Australia (n=13), and the United States (n=9), with a few other studies being set elsewhere in Europe, Southeast Asia, and North America. However, it is noteworthy that the NASSS framework was developed in the United Kingdom, and several included studies were part of the initial empirical testing and refinement of the NASSS domains [25].

**Table 1:** Study characteristics.

Author & Year	Country	Study design	Setting	Study participants	Condition/ Diagnosis	Intervention type	Brief Intervention Description	Timing of the NASSS framework use in implementation	Study design aspects the NASSS framework was used for	NASSS tools used
Abimbola 2019 [26]	Australia	Mixed-Methods	Australian General Practice	Patients; Service providers; Other: program evaluation team (PI, investigators, PhD student)	Cardiovascular related	Digital intervention	Quality improvement intervention for cardiovascular disease prevention & a third-party add-on software tool	Retrospective (to evaluate implementation)	Data analysis; Presentation of results; Interpretation	No tool used
Alhmoud 2022 [27]	England	Qualitative	Hospitals	Service providers; Clinic Staff	Cardiovascular-related	Digital intervention	EHR-integrated automated monitoring devices	Retrospective (to evaluate implementation)	Data collection; Data analysis; Presentation of results; Interpretation	No tool used
Banck 2020 [28]	Sweden	Qualitative	Hospital - Outpatient psychiatric clinics	Service providers; Clinic Staff	Other: Insomnia	Digital intervention	iCBT	Retrospective (to evaluate implementation)	Data collection; Data analysis; Presentation of results; Interpretation	Developed own instrument (based on NASSS)
Barnett 2022 [29]	Australia	Qualitative	Hospital & Ambulatory Care	Service providers; Clinic Staff	Diet & Nutrition: Lifestyle-related chronic conditions	Digital intervention	Technology-supported models of nutrition care	Prospective (to inform design)	Data collection; Data analysis; Presentation of results; Interpretation	No tool used
Bezuidenhout 2022 [30]	Sweden	Quantitative: Observational	Swedish Association of Physiotherapists	Service providers	Neurological diseases, Elderly Care: Older Adults	Digital intervention	Telehealth	Concurrent with implementation	Data collection	No tool used
Brown 2022 [31]	England	Qualitative	Hospitals - acute psychiatric wards	Patients; Clinic Staff	Mental Health: agoraphobic avoidance	Digital intervention	Virtual reality therapy	Prospective (to inform design)	Study design; Data collection; Interpretation	No tool used
Budhwani 2021 [32]	Canada	Qualitative	Hospital - Mental health department	Patients; Service providers	Mental health	Digital intervention	Virtual care (video visit)	Retrospective (to evaluate implementation)	Data analysis	No tool used
Cartledge 2022 [33]	Australia	Qualitative	Members of the Australian Cardiovascular Health and Rehabilitation Association (ACRA)	Service providers	Cardiovascular related: Cardiac event or diagnosis	Digital intervention	Technology use for remotely delivered cardiac rehabilitation	Retrospective (to evaluate implementation)	Data analysis; Presentation of results; Interpretation	No tool used
Catapan 2022 [34]	Brazil	Case study	Hospital & Outpatient Clinic	Service providers; Intervention developers/vendors	Generalized: Patients seeking healthcare during the pandemic	Digital intervention	Teleconsultation	Retrospective (to evaluate implementation)	Data analysis; Presentation of results; Interpretation	No tool used
Clarkson 2020 [35]	United Kingdom	Mixed-Methods	Community organizations	Patients	Pain-related: Joint pain	Digital intervention	Digital self-management tool and social network activation tool	Prospective (to inform design)	Data analysis; Presentation of results; Interpretation	No tool used
Davies 2021 [36]	United Kingdom (Greater Manchester area)	Mixed-Methods	Two schools	Patients; Caregivers; Service providers	No condition specified (school children)	Digital intervention	A reading screening assessment that uses eye-tracking technology and a digital support and well-being monitoring platform	Retrospective (to evaluate implementation)	Data analysis; Presentation of results; Interpretation	No tool used
Dijkstra 2019 [37]	Netherlands	Case study	Hospitals - pediatric gastroenterology centers	Patients; Service providers; Other: Research staff, web designer	Paediatric inflammatory bowel disease	Digital intervention	Web-based telemonitoring strategy	Retrospective (to evaluate implementation)	Study design; Data collection; Data analysis; Presentation of results; Interpretation	No tool used
Dyb 2021 [38]	Norway, Denmark	Qualitative	Various healthcare centres	Service providers; Clinic Staff; Intervention developers/vendors; IT staff; Organizations’ leadership; Government/policymakers	Respiratory Illness: COPD, Elderly care: elderly/frail patients	Digital intervention	Remote patient monitoring, mobile care in patients’ homes, telemedicine	Prospective (to inform design)	Data collection; Data analysis; Presentation of results; Interpretation	Developed own instrument (based on NASSS)
Edridge 2019 [39]	United Kingdom	Quantitative: Experimental	Schools: primary & secondary	Patients; Service providers	Mental health: Children’s mental health	Digital intervention	mHealth: Mental health education	Retrospective (to evaluate implementation)	Data analysis; Interpretation	No tool used
Fox 2021 [40]	Australia	Mixed-Methods	Hospital	Patients; Service providers	Women’s Health: Pregnancy	Digital Intervention	Non-invasive fetal ECG monitoring device	Retrospective (to evaluate implementation)	Data analysis	No tool used
Franck 2021 [41]	USA	Qualitative	Various children’s hospitals	Service providers; Organizations’ leadership	Not condition specific: Various acute illnesses for Medi-Cal beneficiaries	Digital intervention	Rapid genome sequencing	Retrospective (to evaluate implementation)	Data collection	No tool used
Gorbenko 2022 [42]	United States	Qualitative	Healthcare system	Service providers; IT staff; Organizations’ leadership	COVID-19 related	Digital intervention	Google Nest DTC cameras customized for inpatient monitoring.	Prospective (to inform design)	Study design; Data collection; Data analysis; Presentation of results; Interpretation	No tool used
Grady 2020 [43]	Australia	Quantitative: Observational	Various childcare centres	Clinic Staff	Diet & Nutrition: Dietary guidelines	Digital intervention	Digital health interventions to support dietary guideline implementation	Prospective (to inform design)	Data collection; Data analysis; Presentation of results; Interpretation	Developed own instrument (based on NASSS)
Greenhalgh 2018 [44]	United Kingdom	Mixed-Methods	Hospital - various departments	Patients; Service providers; IT staff; Organizations’ leadership; Government/policymakers	Other: Diabetes, Women’s Health: diabetes antenatal, Cancer surgery	Digital intervention	Video outpatient consultations	Concurrent with implementation	Study design; Interpretation	No tool used
Greenhalgh 2018 [25]	United Kingdom	Case study	Healthcare organisations and national-level bodies	Patients; Caregivers (e.g., family members); Service providers; IT staff; Organizations’ leadership; Other: Research staff	Neurological Diseases: Cognitive impairment, Cardiovascular related: heart failure, general data management	Digital intervention	Various technologies: Video outpatient consultations, GPS tracking technology for cognitive impairment, pendant alarm services, remote biomarker monitoring, care organising software, integrated case management	Retrospective (to evaluate implementation)	Data analysis; Presentation of results; Interpretation	No tool used
Gremyr 2020 [12]	Sweden	Case study	Teaching hospital psychiatric department	Service providers; Clinic Staff; IT staff; Organizations’ leadership	Mental Health: Schizophrenia	Digital intervention	Digital Dashboard for Schizophrenia Care	Prospective (to inform design)	Data collection; Data analysis; Presentation of results; Interpretation	NASSS-CAT LONG
Hall 2020 [45]	England	Qualitative	Hospital	Service providers; Clinic Staff; Other: Consultants	Palliative care	Non-digital intervention	Evidence-based Carer Support Needs Assessment Tool to support carers during hospital discharge at end of life.	Retrospective (to evaluate implementation)	Interpretation	No tool used
Hammerton 2022 [46]	England	Mixed-Methods	No condition specified: General practitioner practices	Service providers; Clinic Staff; Intervention developers/vendors	General practice patients	Digital intervention	Various digital healthcare technologies	Prospective (to inform design)	Study design; Data collection	No tool used
Hehakaya 2020 [47]	Netherlands	Qualitative	Hospitals	Patients; Service providers*; Organizations’ leadership; Other: Payers (insurance) & industry	Cancer: Prostate	Digital intervention	MRI-guided radiation therapy	Concurrent with implementation	Data collection; Data analysis	No tool used
Hehakaya 2020 [48]	Netherlands	Qualitative	Hospitals	Patients; Service providers; Organizations’ leadership; Other: Care insurers, manufacturing industry executives	Cancer: Prostate	Digital intervention	MRI-guided radiation therapy	Concurrent with implementation	Data collection; Data analysis	No tool used
Hehakaya 2022 [49]	United States	Qualitative	Hospital- Radiation therapy/radiology departments	Service providers; Clinic Staff; Organizations’ leadership	Cancer	Digital Intervention	MRI-guided radiation therapy	Concurrent with implementation	Data collection	No tool used
Hollick 2019 [50]	United Kingdom (England & Scotland)	Case study	Multiple UK Health Boards- Mobile bone density scanning services	Patients; Service providers; Government/policymakers	Other: Osteoporosis	Digital intervention	Mobile body scanner for bone density	Multiple timepoints	Study design; Data collection; Data analysis; Presentation of results; Interpretation	No tool used
Jacobs 2022 [51]	United States	QT: Observational	Various veteran affairs medical centres	Patients	Not condition-specific	Digital intervention	Telehealth	Retrospective (to evaluate implementation)	Data collection	No tool used
Jones 2022 [52]	United Kingdom	Qualitative	Various social care and volunteer sectors in health settings	Service providers	Occupational therapy treatment for stroke, geriatrics; therapeutics and palliative care (Elderly Care)	Digital intervention	Remote home visits for occupational therapy	Retrospective (to evaluate implementation)	Study design; Data collection; Data analysis; Interpretation	NASSS-CAT LONG
Kip 2020 [53]	Netherlands	Mixed-Methods	Forensic mental healthcare organization	Patients; Service providers	Mental health	Digital intervention	Modules for various topics via a website	Retrospective (to evaluate implementation)	Data collection; Data analysis; Presentation of results; Interpretation	No tool used
Kozica- Olenski 2022 [54]	Australia	Qualitative	General maternity care	Patients; Service providers	Women’s Health: Diabetes in pregnancy	Digital intervention	Telehealth	Retrospective (to evaluate implementation)	Data analysis; Presentation of results; Interpretation	No tool used
Kozica- Olenski 2022 [55]	Australia	Qualitative	Hospital-Menopause Clinic	Patients; Service providers	Women’s Health: Menopause	Digital intervention	Telehealth	Retrospective (to evaluate implementation)	Study design; Data collection; Data analysis; Interpretation	No tool used
Liverani 2022 [56]	Cambodia	Qualitative	Ministry of Health and local and international non-governmental organizations.	Clinic Staff; Government/policymakers; Other: NGOs, WHO	Cardiovascular and other non-communicable diseases	Digital intervention	Wearable health monitors	Prospective (to inform design)	Study design; Data collection; Data analysis; Presentation of results; Interpretation	No tool used
Longacre 2021 [57]	USA	Mixed-Methods	Hospital - Supportive Oncology and Palliative Care Program	Patients; Caregivers	Cancer	Digital intervention	Patient-caregiver portal system	Prospective (to inform design)	Data collection; Data analysis; Presentation of results; Interpretation	No tool used
Martindale 2021 [58]	United Kingdom	Qualitative	Various primary and secondary health care settings	Service providers; Government/policymakers; Other: Scientists	COVID-19 - Related	Non-digital intervention	No intervention; focus on pandemic diagnostic preparedness and testing strategies	Retrospective (to evaluate implementation)	Data analysis	No tool used
Merolli 2019 [59]	Australia	Qualitative	Various clinical health care settings	Patients; Service providers	Pain-related: Chronic low-back pain	Digital intervention	Non specified (“technologies”)	Prospective (to inform design)	Data collection	Developed own instrument (based on NASSS)
Miller 2021 [60]	United Kingdom	Mixed-Methods	Hospital- Stroke specialist staff	Service providers; Clinic Staff**	Cardiovascular related: Stroke	Digital intervention	Online toolkit	Concurrent with implementation	Data analysis; Presentation of results	No tool used
Neher 2022 [61]	Sweden	Qualitative	Four county councils	Patients; Government/policymakers	Cardiovascular related: Heart disease Mental Health: depression	Digital intervention	Four eHealth interventions, including iCBT.	Retrospective (to evaluate implementation)	Interpretation	No tool used
Nguyen 2022 [62]	United States	Quantitative: Experimental	Home-based care - various sites	Patients; Caregivers; Service providers	Palliative care, Elderly Care: aging, Not condition specific: various serious illness with an expected survival of 1–2 year	Digital intervention	Video consultation	Concurrent with implementation	Data analysis; Presentation of results; Interpretation	No tool used
Nimsakul 2022 [63]	Thailand	Other	Hospitals	Service providers; Clinic Staff; Organizations’ leadership; Other: civil society member, experts in drug operations	Mental Health: Harm reduction	Non-digital intervention	Harm reduction service	Retrospective (to evaluate implementation)	Data collection; Data analysis; Presentation of results	No tool used
Papoutsi 2020 [13]	United Kingdom	Qualitative	Hospitals & Primary care	Patients; Caregivers (e.g., family members); Service providers; Other: researchers	Cardiovascular related: Heart failure	Digital intervention	Various tools	Retrospective (to evaluate implementation)	Data collection; Data analysis; Presentation of results	No tool used
Perdacher 2022 [64]	Australia	Qualitative	Prisons	Patients; Service providers	Mental Health	Digital intervention	Digital mental health tool	Retrospective (to evaluate implementation)	Interpretation	No tool used
Przysucha 2022 [65]	Germany	Qualitative	Nursing care facilities & GP practices	Service providers	Not condition-sympathetic: Primary care	Digital intervention	eMedCAre	Retrospective (to evaluate implementation)	Interpretation	No tool used
Pumplun 2021 [66]	Germany	Qualitative	Various clinics	Service providers; Clinic Staff; Other: “highly involved experts” who have detailed knowledge of clinical processes, experience with ML systems, and are involved in the respective decision-making processes: clinics’ managers, physicians, and managers of diagnostic HIT suppliers.	Not condition-specific	Digital intervention	Understanding of clinics’ adoption process of ML system	Prospective (to inform design)	Data collection; Data analysis; Presentation of results; Interpretation	No tool used
Pumplun 2021 [67]	Germany & Switzerland	Qualitative	Various clinics	Service providers; Clinic Staff; IT staff; Organizations’ leadership	Machine learning- medical diagnosis	Digital intervention	Machine Learning systems for medical diagnostics in clinics	Prospective (to inform design)	Data analysis; Presentation of results; Interpretation	No tool used
Rudin 2021 [68]	United States	Other: Multi-methods	Various primary care clinics affiliated with an academic health system	Patients; Service providers	Respiratory Illness: Asthma	Digital intervention	Clinically integrated remote symptom monitoring intervention	Prospective (to inform design)	Data collection; Data analysis; Presentation of results; Interpretation	No tool used
Schougaard 2019 [69]	Denmark	Quantitative: Experimental	Hospital - Department of Neurology	Patients	Neurological Diseases: Epilepsy	Digital intervention	Telehealth & website	Retrospective (to evaluate implementation)	Interpretation	No tool used
Schultz 2021 [70]	Australia	Mixed-Methods	Hospital - virtual ward	Patients; Caregivers; Service providers	COVID-19- Related	Digital intervention	Virtual hospital ward	Concurrent with implementation	Data collection	NASSS-CAT LONG
Strohm 2020 [71]	Netherlands	Case study	Hospitals - Radiology departments	Service providers; Clinic Staff; Organizations’ leadership; Other: Innovation manager, senior data scientist	Other: Radiology	Digital intervention	AI applications in clinical radiology	Retrospective (to evaluate implementation)	Data collection; Data analysis; Presentation of results; Interpretation	No tool used
Thomas 2022 [72]	Australia	Qualitative	Various state-wide cardiac and pulmonary networks	Service providers; Clinic Staff	Cardiovascular related: Cardiopulmonary health	Digital intervention	Telehealth	Retrospective (to evaluate implementation)	Data collection; Data analysis; Presentation of results; Interpretation	No tool used
Thomas 2022 [72]	Australia	Other: Multi-Method	Metropolitan health service network	Service providers; Clinic Staff; Other: allied health departments	Not condition-specific	Digital intervention	Telehealth	Retrospective (to evaluate implementation)	Data collection; Data analysis; Presentation of results; Interpretation	No tool used
Tolf 2020 [73]	Sweden	Qualitative	Hospital - Obstetric unit	Service providers; Clinic Staff	Women’s Health: Obstetrics and gynecology	Digital intervention	Technology-supported QI programme	Retrospective (to evaluate implementation)	Data collection; Data analysis; Presentation of results	No tool used
Tompson 2019 [74]	United Kingdom	Mixed-Methods	General Practitioner Surgery Clinics	Patients; Service providers	Cardiovascular related: Hypertension	Digital intervention	Blood pressure self-measurement kiosks	Retrospective (to evaluate implementation)	Interpretation	No tool used
Uribe Guajardo 2022 [75]	Australia	Other: Multi methods	Outpatient drug and alcohol services	Service providers	Mental Health: Comorbid mental health and substance use problems	Digital intervention	Portal with eHealth Resources	Retrospective (to evaluate implementation)	Data analysis; Presentation of results; Interpretation	No tool used
Vali, 2022 [76]	Various European countries	Mixed-Methods	International setting	Service providers	Other: Non-alcoholic fatty liver disease (NAFLD)	Non-digital intervention	Various non-alcoholic fatty liver disease non-invasive tests	Prospective (to inform design)	Data collection; Data analysis; Presentation of results	No tool used
Weidner 2021 [77]	United States	Mixed-Methods	International - various twitter user groups	Service providers; Other: Twitter users including users from nonprofit organization, public (personal account and/or health care consumer), business (for-profit group) ad unknown	Not condition-specific	Digital intervention	Telepractice used by speech language pathologists	Retrospective (to evaluate implementation)	Data collection; Data analysis; Presentation of results	No tool used
Yakovchenko 2021 [78]	United States	Qualitative	Various medical centres	Patients; Service providers	Not condition specific: Veterans’ Health and Wellness	Digital intervention	Automated texting system	Retrospective (to evaluate implementation)	Data collection; Data analysis; Presentation of results; Interpretation	Other: NASSS-CAT LONG

*Service providers includes physicians and therapists.

**Clinic staff include clinic administrators and managers.

**Table 2:** Summary characteristics.

Characteristic			Citation
**Study Type**	**n**	**%**	
*Qualitative*	28	49.1	[13,27–29,31–33,38,41,42,45,47–49,52,54–56,58,59,61,64–67,72,73,78]
*Mixed-methods*	13	22.8	[26,35,36,40,44,46,53,57,60,70,74,76,77]
*Case Study*	6	10.5	[12,25,34,37,50,71]
*Observational*	3	5.3	[30,43,51]
*Experimental*	3	5.3	[39,62,69]
*Other*	4	7.0	[63,68,75,79]
**Country of Origin**	**n**	**%**	
*United Kingdom (including studies set in individual UK countries and across the UK)*	15	26.3	[13,25,27,31,35,36,39,44–46,50,52,58,60,74]
*Australia*	13	22.8	[26,29,33,40,43,54,55,59,64,70,72,75,79]
*USA*	9	15.8	[41,42,49,51,57,62,68,77,78]
*Sweden*	5	8.8	[12,28,30,61,73]
*the Netherlands*	5	8.8	[37,47,48,53,71]
*Germany*	2	3.5	[65,66]
*Thailand*	1	1.8	[63]
*Brazil*	1	1.8	[34]
*Denmark*	1	1.8	[69]
*Canada*	1	1.8	[32]
*Cambodia*	1	1.8	[56]
*Multiple countries*	3	5.3	[38,67,76]
Conditions Studied*	**n**		
*Cardiovascular-related*	10		[13,25–27,33,56,60,61,72,74]
*Mental Health*	9		[12,31,32,39,53,61,63,64,75]
*Generalized*	9		[25,34,41,46,62,65–67,78]
*No condition specified*	5		[36,51,67,77,79]
*Cancer*	5		[44,47–49,57]
*Women’s health*	5		[40,44,54,55,73]
*Elderly care*	4		[30,38,52,62]
*Neurological disease*	3		[25,30,69]
*COVID-related*	3		[42,58,70]
*Pain-related*	2		[35,59]
*Diet and Nutrition*	2		[29,43]
*Respiratory Illness*	2		[38,68]
*Palliative care*	2		[45,62]
Other**	6		[28,37,44,50,71,76]

*Not mutually exclusive.

**Other conditions include diabetes, insomnia, non-alcoholic fatty liver disease, osteoporosis, radiology.

Since the NASSS framework was designed for health technology innovations, there were a variety of health conditions for which innovations were implemented, including cardiovascular (n=10), mental health (n=9), general health promotion (n=9), cancer (n=5), and women’s health (n=5), among others. Of the 57 included studies, 53 implemented digital applications, and the rest (n=4) implemented non-digital interventions, such as harm reduction services and COVID-19 testing strategies. Of the 53 digital applications, approximately half of them were virtual care, which included telemedicine (n=24), followed by personal health devices (n=10), knowledge generation applications (n=9), and digital interventions (n=10), such as internet-based Cognitive Behavioural Therapy (iCBT). See Table 3 for a complete list of digital applications and examples.

**Table 3:** Innovations examined by included studies.

*Category	Example of Interventions	n*	%	Citations
Virtual Care	Telemedicine, eHealth virtual care & monitoring	24	34.78	[13,25,28,30,32–34,37,38,42,44,46,51,52,54,55,57,61,62,68,70,72,77,79]
Personal Health Devices	Self-management/monitoring tools, self-assessment	10	14.49	[13,25,35,36,40,46,56,65,74,78]
Knowledge Generation and/or Integrators	eLearning, machine learning, decision aids, tools (web or app based)	9	13.04	[25–27,60,66,67,71,73,75]
Digital intervention	A single intervention/application that is digital and doesn’t fit the above categories. (e.g., iCBT, VR therapy)	10	14.49	[28,30,31,33,39,43,46,53,64,69]
Health Information	EMRs/patient records dashboards, patient portals	8	11.59	[12,13,46,57,65,69,73,75]
Surgical & Radiologic Interventions	Radiotherapy or new surgical intervention	4	5.80	[47–49,71]
Diagnostics & Imaging	Interventions that conduct diagnostic testing or imaging, onsite or remote.	2	2.90	[41,50]
Non-specified	Study just states “technologies” or “interventions”	2	2.90	[29,59]

**Note**: Categories adapted based on “Evolving Applications of Digital Technology in Health and Health Care” as cited in Abernethy et al., 2022 [24].

*Not mutually exclusive.

### RQ2. Application of the NASSS framework

As indicated in Table 4, the NASSS framework was used in various aspects of the study methodology. The NASSS framework was used to inform the overall study design (n=9), including conceptualization. Studies used the NASSS framework to inform data collection methods (n=35) by adapting interview guides according to the NASSS domains (e.g., [47,72]). Studies also used the NASSS framework to inform data analysis (n=41), for example, using the NASSS framework for directed content analysis (e.g., [66]). The NASSS framework was also used to inform data presentation (n=33) such as utilizing a table to organize barriers and enablers by NASSS domains (e.g., [54]). Finally, studies used the NASSS framework to interpret results (n=39), for example, by dedicating one paragraph of the discussion to each NASSS domain (e.g., [61]). Most papers (n=43) used the NASSS framework to inform multiple aspects of their study.

**Table 4:** Application of the NASSS framework.

NASSS Framework Application Characteristic	n	%	Citation
Study Design Aspect*			
*Overall Study Design*	9	5.7	[31,37,42,44,46,50,52,55,56]
*Data Collection*	35	22.3	[12,13,27–31,37,38,41–43,46–53,55–57,59,63,67,68,70–73,76–79]
*Data Analysis*	42	26.1	[12,13,25–29,32–40,42,43,47,48,50,52–58,60,62,63,66–68,71–73,75–79]
*Presentation of results*	34	21.0	[12,13,25–29,33–38,42,43,50,53,54,56,57,60,62,63,66–68,71–73,75–79]
*Interpretation of results*	39	24.8	[12,25–29,31,33–39,42–45,50,52–57,61,62,64–69,71,72,74,75,78,79]
**Timing of Implementation**			
*Retrospectively*	33	57.9	[13,25–28,32–34,36,37,39–41,45,51–55,58,61,63–65,69,71–75,77–79]
*Prospectively*	15	26.3	[12,29,31,35,38,42,43,46,56,57,59,66–68,76]
*Concurrent with Implementation*	8	14.0	[30,44,47–49,60,62,70]
*Multiple Time Points*	1	1.8	[50]
**Number of NASSS domains reported**			
*1 domain*	1	1.75	[59]
*2 domains*	1	1.75	[77]
*3 domains*	3	5.26	[43,69,70]
*4 domains*	11	19.30	[27,30,31,35,39,42,46,60,65,74,75]
*5 domains*	9	15.79	[32,34,41,45,48,51,58,61,64]
*6 domains*	13	22.81	[29,44,47,49,52,53,55,57,66–68,71,78]
*7 domains*	19	33.33	[12,13,25,26,28,33,36–38,40,50,54,56,62,63,72,73,76,79]

*Not mutually exclusive.

Regarding timing, most studies conducted their analyses using the NASSS framework retrospective to implementation, for example, to analyze why implementation succeeded or failed to support adoption, non-abandonment, scale, spread, and sustainability of the innovation within a given context (n=33). The rest applied the framework prospectively to inform future implementations (n=15) or concurrently with implementation (n=8). Approximately one-third (32%) of included studies reported implementation barriers and enablers related to all 7 NASSS domains, and 21% reported barriers and enablers related to 6 domains. The Embedding and Adaptation Over Time domain was often omitted, but studies incorporated this concept into other domains (e.g., whether the technology will require future iterations [27], whether the regulatory context is expected to change [41]). Another one-third (35%) of studies reported barriers and enablers related to four to five NASSS domains, while 12% reported three or fewer domains. The latter relied on advisory committees to identify domains relevant to the study [43].

**Fig 2:**
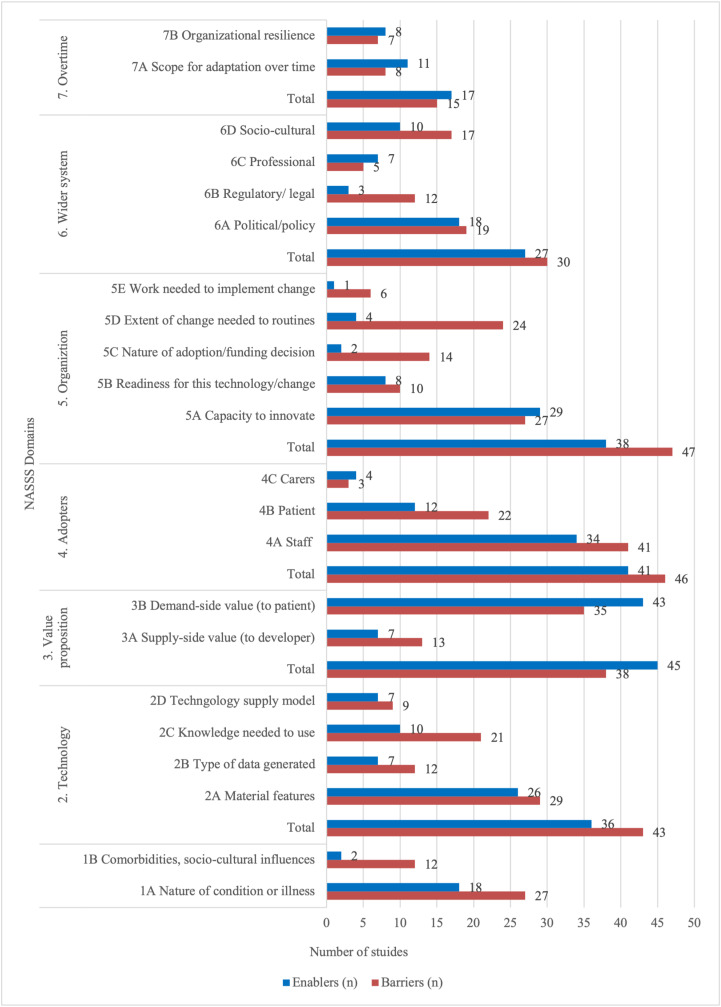
Barriers and Enablers identified in included studies, organized according to NASSS domains.

Studies often identified implementation determinants using the NASSS framework. The barriers and enablers of the successful implementation of innovations are presented by the NASSS domain in Fig 2. The most common barriers across studies (n=47) were in the Organization domain, where organizations were cited as lacking infrastructure, resources, or capacity to innovate and/or where the innovation substantially disrupted organizational routines. Specifically, organizational capacity, such as technical or human resources, was the most frequently reported barrier. Another common barrier within the Organization domain was the extent of change required in routine practice. The following are some exemplary quotes of organizational barriers reported in studies:

*“Technical infrastructure was sometimes poor, increasing the likelihood of technical crashes.”* [26]

*“Representatives from all three groups expressed that an impediment to engaging in the [Quality Improvement] teams was insufficient time and that meeting times conflicted with clinical engagements.”* [73]

*“Space and the need for dedicated and private telehealth rooms were also common concerns for clinicians. Such spaces need to be fitted with appropriate hardware, software, and peripheral devices.”* [72]

*“Therapists stated that the intervention was often not discussed in meetings and was not integrated in electronic patient records they used.”* [53]

*“Participants indicated they were concerned that administrative tasks would continue to be a significant time barrier with increased adoption and scale up.”* [29]

The most reported enablers were within the Value Proposition domain. A total of 45 studies noted the technology as profitable (from the supply side) or cost-effective (from the demand side) and reported perceived advantages, including improved patient outcomes, increased access to care, enhancements in organizational processes or workflows, and overall effectiveness of the innovation. The following are exemplar quotes of enablers related to the Value Proposition domain reported in studies:

*“With automated monitoring in the specialist hospital, the accuracy of recording and timely data transfer is reliable. Nurses are more aware of the need to accomplish this task when it’s automated.”* [27]

*“Clinicians valued telehealth for the benefits they felt it afforded patients such as convenience and improved access to care, more so than perceived advantages for themselves.”* [55]

*“Several practical advantages were mentioned, among which saving time for therapists and patients because of less traveling time and replacing part of in-person treatment with the intervention, an increase of patients’ access to care because they can individually work on their treatment at their own pace, and providing a new way of delivering treatment to patients.”* [53]

Factors within the Adopter System domain were also commonly reported as barriers or enablers to implementation. A total of 46 studies reported Adopter System factors as barriers and 41 studies reported them as enablers. These included the attitudes and acceptance of staff, patients, and carers towards the new technology and its ease of use. Notably, staff was more frequently reported than patients as both a barrier and an enabler. The following are some exemplar quotes of barriers and enablers related to the Adopter System domain reported in studies:

*“A few therapists were willing to try ICBT-i, but none were initially deeply interested in the new method, only a few were available to take on this extra task, and only a few had the appropriate competence.”* [28]

*“Lastly, providers described feelings of `Zoom fatigue’ and burnout and mentioned that video visits required more concentration, energy, and adaptations to interpret visual cues in comparison to in-person visits.”* [32]

*“Most patient participants were interested to see their readings and described the technology as well-designed. They used the tablet and the peripheral devices without too much difficulty and saw great value in monitoring their condition, especially in terms of gaining reassurance and legitimising help-seeking when they needed clinical care.”* [13]

### RQ3. Lessons learned from the application of the NASSS framework

A few authors (i.e., 25 studies) reported lessons learned from applying the NASSS framework in their studies. Such information had varying levels of detail, often with minimal elaboration. Eighteen studies [12,26,28,33,35,39,41,50,52,55–57,62,63,68,73,75,78] recognized the NASSS framework as a valuable and versatile tool, with researchers explicitly noting its utility in various aspects. These studies highlighted how the NASSS framework effectively aids in exploring the complexities of implementation processes, allowing for a deeper understanding of the intricate contexts in which health technologies are introduced. Additionally, the framework was praised for its applicability across different domains within health technology, proving to be particularly useful in navigating the multifaceted challenges of technology adoption and integration. Furthermore, many researchers appreciated its flexibility, which allowed them to adapt the framework to meet the specific needs of their studies, demonstrating its adaptability and relevance to diverse research objectives. For example, Uribe Guajardo et al. [75] highlighted the flexibility of the NASSS framework regarding the timing of application, noting its effectiveness in retrospective analyses and its ability to draw conclusions about the implementation and long-term sustainability of portal and eHealth resources. The authors also suggested that future research and program design would benefit from using the NASSS framework prospectively, especially with new and revised e-resources [75]. A few studies mentioned the comprehensiveness of the tool for identifying implementation determinants and its value in providing a theoretical foundation [12,27,38]. Additionally, two studies [50,78] suggested future directions for the NASSS framework, highlighting the potential opportunity to use the NASSS-CAT tool over time and to explore its applicability in a broader healthcare context. Lastly, two studies [62,67] commented on the limitation of the NASSS framework, noting its lack of consideration for how research design can impact intervention implementation and the need for its expansion to include medical ethics.

## Discussion

This scoping review identified 57 empirical studies that used the NASSS framework from its publication in August 2017 until the search commenced in December 2022. Most included studies were qualitative or mixed/multi-methods designs, which can be attributed to the purpose of the NASSS framework in exploring determinants of implementation success. This exploration required substantial contextual information, which qualitative data could effectively provide. The NASSS framework was commonly used to inform data collection, data analysis, and the presentation of results. Almost all included studies focused on technological innovation, such as telemedicine, virtual care, health monitoring or decision support devices and applications, and targeted digital interventions. These innovations were designed for various health conditions, primarily cardiovascular and mental health, or supported general health promotion activities. While approximately one-third of studies reported barriers and enablers for implementation on all 7 NASSS domains, 20% did not report barriers or enablers related to the Over Time domain. The most reported barriers were found in the Organization and Adopter System domains, and the most frequently reported enablers were within the Value Proposition domain.

Most studies in this review used the NASSS framework retrospectively, primarily to evaluate why an innovation failed to be adopted by its intended users, was abandoned shortly after implementation, or did not scale to become routine within the organization, spread to other contexts, or sustain over time. Similar findings have been reported with the i-PARiHS (Integrated Promoting Action on Research Implementation in Health Services) application in research [80]. There is a need for prospective and concurrent applications of implementation TMFs to identify potential hurdles and areas of complexity ahead of implementation so that mitigation strategies can be applied [81,82]. Given the novelty of the NASSS framework, many innovations in this review were implemented in small-scale demonstration projects or as larger implementation studies not informed *a priori* by any theoretical framework and, therefore, required retrospective evaluation. Nevertheless, the NASSS framework does not offer solutions to identified areas of complexity. While some authors noted that the NASSS framework helped illuminate areas of focus, it remained unclear what actions they intended to take [26]. A companion document (i.e., NASSS-CAT) [11] explicitly recommends the next steps for each domain where complexity is identified; however, only four included studies had used any part of the NASSS-CAT tool [12,52,70,78].

The prevalent implementation determinants (i.e., barriers and enablers) identified in the Organization and Adopter System domains in this review are consistent with findings from previous reviews of other TMFs used in implementation science. The Exploration, Preparation, Implementation, Sustainment (EPIS) [83] is a commonly used framework that highlights key phases guiding implementation as well as factors related to the outer (system) context, inner (organizational) context and the innovation itself. A review of EPIS showed that the Implementation phase was the most commonly examined in research. In this phase, organizational and individual adopter characteristics were the most frequently mentioned factors [84], similar to what we observed in the current NASSS framework review.

In the dynamic field of implementation science, various determinant frameworks serve a similar role in facilitating the understanding of complex factors, focusing on contextual elements that influence the successful implementation of healthcare innovations. The CFIR, a popular determinant framework in implementation science, primarily identifies factors influencing implementation outcomes across the domains of Intervention Characteristics, Outer Setting, Inner Setting, Individual Characteristics, and Implementation Process [85]. The CFIR serves a similar purpose to the NASSS framework. A recent literature review of the CFIR indicated that the most commonly used constructs in studies were “Knowledge and Beliefs about the Intervention,” followed by “Self-Efficacy,” both of which fall within the CFIR domain of Individual Characteristics [86]. This finding echoes the NASSS Adopter System and the Value Proposition domains that are commonly reported barriers and enablers identified in this current review.

The I-PARiHS is another implementation determinant framework with four interacting core constructs: Evidence, Context, Recipients, and Facilitation [87,88]. The inner and outer Contexts in the i-PARiHS mirror the Organization and Wider Context domains of the NASSS. A review of research studies using the i-PARiHS [89] identified variations in how researchers conceptualized outer Context, including specific influences from external organizations, such as guideline-producing entities, and broader political and economic characteristics attributed to “contextual trust” [89]. This conceptualization resonates with the Wider Context of the NASSS framework. Furthermore, leadership was suggested as another key sub-construct within the Context of the i-PARiHS [89], which corresponds to the 5A Capacity subdomain within the Organization domain of the NASSS framework. Although the NASSS framework was initially created to implement health and care technologies, it exhibits similarities with widely used implementation determinant frameworks designed for a broader range of health innovations, including health technology and evidence-based practices. As such, we found four studies in this review that used the NASSS framework for non-digital innovations [45,58,63,76], demonstrating the framework’s adaptability and utility.

Our review found that, when used, the NASSS framework informed many design aspects, including data collection, analysis, presentation and interpretation. The use of the NASSS framework in data collection and analysis was usually consistently and clearly reported. However, consistency and clarity were lacking when it was used to present and interpret results. Often, data were presented within the primary domains of the NASSS framework. We observed several overlaps as our team organized narrative descriptions of barriers and enablers by NASSS subdomains. Furthermore, we identified the potential for these barriers to be mapped onto other primary NASSS domains. This observation may indicate the intricate nature of the implementation under examination in the included studies, which could be explained by the framework’s underlying assumption that, in complex situations, the NASSS domains interact with one another and are interdependent [25]. In other words, when interdependencies among the domains exist, it often leads to the inability to address a singular issue without inadvertently giving rise to new challenges in other domains of the NASSS [25].

For studies that did not present their results within the NASSS domains, even though the authors reported using the NASSS framework for data analysis, it was challenging to determine which domain(s) the results pertained to in predicting or explaining implementation success or failure. This unclear use of the NASSS framework for presenting results and interpreting findings represents a notable gap in the literature. The literature has previously documented that implementation studies lack reporting, leading to low-quality reporting in the field [90,91]. Specifically, many implementation studies have faced criticism for providing inaccurate context descriptions and lacking detailed information on the implementation process [91]. Poor reporting makes it difficult to synthesize evidence from relevant studies [90]. Therefore, enhancing reporting practices to facilitate more straightforward evidence synthesis is essential, aiding future empirical testing and refinement of the NASSS framework.

Additionally, some studies were unclear about how the NASSS framework was used to inform the overall study designs. Clear reporting standards may increase the utility of the NASSS framework by guiding researchers on correctly applying and describing its use. The need for better reporting on how TMFs are used in implementation research is a gap in the literature that has already been discussed [92]. For example, a review identified 159 different implementation TMFs, of which 87% of them were used in five or fewer studies [92]. Despite the substantial number of TMFs, there is limited evidence to describe their use [92]. This limitation restricts opportunities for advancing the science and learning from other researchers. Implementation studies should report more clearly on how TMFs have been incorporated into the study design [93]. Better reporting allows for a coherent synthesis of evidence, application and scaling of the TMFs to other contexts, thereby contributing to the implementation science [93]. We also found that not many authors shared their experience of using the NASSS framework or provided suggestions for its advancement. Two studies in this review mentioned the shortcomings of the NASSS framework [62,67], including ethical principles, which were addressed in the Planning and Evaluating Remote Consultation Services framework in 2021 [94]. It would be beneficial to conduct a review in five years to reassess the application of the NASSS framework, explore grey literature, and gather lessons learned for the ongoing advancement and refinement of the framework.

Reporting issues have led to the creation of reporting checklists in other fields, such as the Consolidated Standards of Reporting Trials (CONSORT) checklist for randomized controlled trials [95]. Some implementation reporting standards are available; one example is the Standards for Reporting Implementation Studies (StaRI) Statement and Checklist [91]. The StaRI checklist prompts authors to describe the implementation method and the intervention [91], encouraging detailed reporting on contextual information. In addition, the StaRI checklist also prompts authors to describe the theoretical underpinnings of the study. Therefore, its use in future implementation studies is encouraged and may improve reporting of TMF applications, including the NASSS framework.

### Limitations

Several limitations of this review must be acknowledged. First, quality appraisal was not employed to exclude studies, as scoping reviews generally do not require such assessment. In addition, our primary goal was to explore the breadth and depth of the literature and map available literature about the NASSS framework application. Second, the field of mHealth is rapidly evolving, and our findings may need re-evaluation. Nevertheless, our review remains relevant at the time of publication and contributes to the ongoing evolution of the NASSS framework. Third, this review excluded non-empirical papers, such as commentaries and opinion articles, which could offer authors’ insights regarding their experiences with the NASSS framework. Future reviews aiming to reassess the NASSS application can include grey literature to enhance comprehensiveness. Fourth, we only included studies written in English. While we did include a small number of English studies published in non-English speaking countries, our findings may not provide a comprehensive representation of the NASSS framework’s application in those regions.

## Conclusions

This review outlines the characteristics of studies using the NASSS framework and examines patterns of its application. Most of the included studies employed qualitative or mixed/multi-methods designs, which aligns with the NASSS framework’s purpose of exploring determinants of implementation success. This often requires qualitative exploration to assess context. Additionally, most studies retrospectively applied the NASSS framework, likely due to the novelty of the framework. However, this highlights the need for prospective and concurrent utilization of the NASSS framework during the implementation phase, revealing a gap in the current literature. Furthermore, nearly all included studies identified various domains as both implementation barriers and enablers, which aligns with the current literature describing the intricate nature of the implementation process. This underscores the importance of thorough preparation for achieving successful implementation outcomes [96]. Lastly, our review findings highlight the need for improved reporting on the use of the NASSS framework in research, including how it was applied, as well as the need for greater consistency in presenting results and interpreting findings using the NASSS framework to facilitate evidence synthesis in the future.

## Supporting information

S1 Appendix Search strategy. (DOCX)

S2 Appendix Data extraction tool. (DOCX)
