# Supplementary material for: The NASSS (Non-Adoption, Abandonment, Scale-Up, Spread and Sustainability) framework use over time: A scoping review
Source: PLOS Digit Health. 2025 Mar 17;4(3):e0000418. doi: 10.1371/journal.pdig.0000418 (PMC11913280; doi:10.1371/journal.pdig.0000418)
Supplement: S1 Appendix — (DOCX) [file pdig.0000418.s001.docx]

S1 Appendix. Search Strategy

**(Search Ran 20 December 2022)**

**Medline (Ovid)**

Database Ovid MEDLINE: Epub Ahead of Print, In-Process & Other Non-Indexed Citations, Ovid MEDLINE® Daily and Ovid MEDLINE® <1946-Present>

Search strategy:

--------------------------------------------------------------------------------

1 NASSS.ti,ab,kf. (51)

2 ((non-adoption or nonadoption) adj2 abandonment adj5 (scale-up or scaleup) adj2 spread adj2 sustainability).ti,ab,kf. (53)

3 NASSS-CAT.ti,ab,kf. (2)

4 (greenhalgh* adj5 (framework* or model*)).ti,ab,kf. (26)

5 1 or 2 or 3 or 4 (88)

**EMBASE (Ovid)**

Database: Embase Classic+Embase 1947 to 2022 December 19

Search strategy:

--------------------------------------------------------------------------------

1 NASSS.ti,ab,kf. (54)

2 ((non-adoption or nonadoption) adj2 abandonment adj5 (scale-up or scaleup) adj2 spread adj2 sustainability).ti,ab,kf. (51)

3 NASSS-CAT.ti,ab,kf. (2)

4 (greenhalgh* adj5 (framework* or model*)).ti,ab,kf. (35)

5 1 or 2 or 3 or 4 (97)

**APA PsychInfo (Ovid)**

Database: APA PsycInfo 1806 to December Week 2 2022

Search strategy:

--------------------------------------------------------------------------------

1 NASSS.ti,ab,id. (12)

2 ((non-adoption or nonadoption) adj2 abandonment adj5 (scale-up or scaleup) adj2 spread adj2 sustainability).ti,ab,id. (6)

3 NASSS-CAT.ti,ab,id. (0)

4 (greenhalgh* adj5 (framework* or model*)).ti,ab,id. (8)

5 1 or 2 or 3 or 4 (22)

**CINAHL (EBSCO)**

Database: CINAHL Plus with Full Text

Search strategy:

--------------------------------------------------------------------------------

1 TI NASSS OR AB NASSS OR TX NASSS (34)

2 TI ( ((non-adoption OR nonadoption) N2 abandonment N2 (scale-up or scaleup) N2 spread N2 sustainability) ) OR AB ( ((non-adoption OR nonadoption) N2 abandonment N2 (scale-up or scaleup) N2 spread N2 sustainability) ) OR TX ( ((non-adoption OR nonadoption) N2 abandonment N2 (scale-up or scaleup) N2 spread N2 sustainability) ) (20)

3 TI NASSS-CAT OR AB NASSS-CAT OR TX NASSS-CAT (3)

4 TI ( greenhalgh* N5 (framework* OR model*) ) OR AB ( greenhalgh* N5 (framework* OR model*) ) OR TX ( greenhalgh* N5 (framework* OR model*) ) (74)

5 S1 OR S2 OR S3 OR S4 (109)

**LISTA (EBSCO)**

Database: Library, Information Science & Technology Abstracts

Search strategy:

--------------------------------------------------------------------------------

1 TI NASSS OR AB NASSS OR TX NASSS (9)

2 TI ( ((non-adoption OR nonadoption) N2 abandonment N2 (scale-up or scaleup) N2 spread N2 sustainability) ) OR AB ( ((non-adoption OR nonadoption) N2 abandonment N2 (scale-up or scaleup) N2 spread N2 sustainability) ) OR TX ( ((non-adoption OR nonadoption) N2 abandonment N2 (scale-up or scaleup) N2 spread N2 sustainability) ) (9)

3 TI NASSS-CAT OR AB NASSS-CAT OR TX NASSS-CAT (1)

4 TI ( greenhalgh* N5 (framework* OR model*) ) OR AB ( greenhalgh* N5 (framework* OR model*) ) OR TX ( greenhalgh* N5 (framework* OR model*) ) (1)

5 S1 OR S2 OR S3 OR S4 (12)

**Web of Science**

Database: Web of Science Core Collection (1900-present)

Search strategy:

--------------------------------------------------------------------------------

1 TS=(NASSS) (48)

2 TS=((non-adoption or nonadoption) NEAR/2 abandonment NEAR/5 (scale-up or scaleup) NEAR/2 spread NEAR/2 sustainability ) (49)

3 TS=(NASSS-CAT) (2)

4 TS=(greenhalgh* NEAR/5 (framework* OR model*)) (30)

5 #1 OR #2 OR #3 OR #4 (89)

**Scopus**

Database: Scopus

Search strategy:

--------------------------------------------------------------------------------

1 TITLE-ABS-KEY ( nasss ) (55)

2 TITLE-ABS-KEY ( ( non-adoption OR nonadoption ) W/2 abandonment W/5 ( scale-up OR scaleup ) W/2 spread W/2 sustainability ) (43)

3 TITLE-ABS-KEY ( nasss-cat ) (2)

4 TITLE-ABS-KEY ( greenhalgh* W/5 ( framework* OR model* ) ) (38)

5 TITLE-ABS-KEY ( nasss ) ) OR ( TITLE-ABS-KEY ( ( non adoption OR nonadoption ) W/2 abandonment W/5 ( scale-up OR scaleup ) W/2 spread W/2 sustainability ) ) OR ( TITLE-ABS-KEY ( nasss-cat ) ) OR ( TITLE-ABS-KEY ( greenhalgh* W/5 ( framework* OR model* ) ) ) (103)
