# Supplementary material for: The NASSS (Non-Adoption, Abandonment, Scale-Up, Spread and Sustainability) framework use over time: A scoping review
Source: PLOS Digit Health. 2025 Mar 17;4(3):e0000418. doi: 10.1371/journal.pdig.0000418 (PMC11913280; doi:10.1371/journal.pdig.0000418)
Supplement: S2 Appendix — (DOCX) [file pdig.0000418.s002.docx]

|  | | Value | |
| --- | --- | --- | --- |
| Citation information | Author |  | |
|  | Year |  | |
|  | Study title |  | |
|  | Link |  | |
|  | Journal |  | |
| Study characteristics | Type of source |  | |
|  | Country (setting) |  | |
|  | Study timeframe |  | |
|  | Study aims |  | |
|  | Study design (e.g., cross-sectional, retrospective cohort) |  | |
|  | Data sources | Select all:   - Health-admin data - Survey - Interview - Focus Group - Other : Insert text | |
|  | Setting |  | |
|  | Study participants (data collected from them for the purpose of the study aim) |  | |
|  | Condition/diagnosis |  | |
|  | Intervention type |  | |
|  | Intervention (describe) |  | |
|  | Intervention targets (e.g., patients with a specific condition) |  | |
| How NASSS was applied | Timing of framework use with regards to implementation | Select:   - Prospective - Retrospective - Concurrently | |
|  | NASSS was used in: | Select all:   - Study design - Data collection - Analysis - Presentation - Interpretation | |
|  | Were NASSS tools used? |  | |
| Barriers (NASSS domains) | Description of barriers |  | |
|  |  | Select corresponding sub domains (can select multiple) | |
|  | (1) The illness/condition | - 1A. Nature of Condition/Illness | |
|  |  | - 1B. Comorbidities | |
|  |  | - 1C. Sociocultural factors | |
|  |  | - The illness/condition: other (list) | |
|  |  | - 2A. Material properties | |
|  | (2) The technology | - 2B. Knowledge to use | |
|  |  | - 2C. Knowledge generated | |
|  |  | - 2D. Supply model | |
|  |  | - 2E. Who owns the IP | |
|  |  | - The technology: other (list) | |
|  | (3) The value proposition | - 3A. Supply-side value (to developer) | |
|  |  | - 3B. Demand-side value (to patient) | |
|  |  | - The value proposition: other (list) | |
|  | (4) The adopters | - 4A. Staff (role, identity) | |
|  |  | - 4B. Patient (passive vs. active input) | |
|  |  | - 4C. Carers (available, type of input) | |
|  |  | - The adopters: other (list) | |
|  | (5) The organization(s) | - 5A. Capacity to innovate | |
|  |  | - 5B. Readiness for this technology | |
|  |  | - 5C. Nature of adoption/funding decision | |
|  |  | - 5D. Extent of changes needed to organisational routines | |
|  |  | - 5E. Work needed to implement and evaluate change | |
|  |  | - The organization: other (list) | |
|  | (6) The wider system | - 6A. Political/policy context | |
|  |  | - 6B. Regulatory/legal issues | |
|  |  | - 6C. Professional bodies | |
|  |  | - 6D. Socio-cultural context | |
|  |  | - 6E. Inter-organisational networking | |
|  |  | - The wider system: Other (list) | |
|  | (7) Over time | - 7A. Scope for adaptation over time | |
|  |  | - 7B. Organisational resilience | |
|  |  | - over time: other (list) | |
| Enablers (NASSS domains) | Description of enablers |  | |
|  | (1) The illness/condition | Select corresponding sub domains (can select multiple) | |
|  |  | - 1A. Nature of Condition/Illness | |
|  |  | - 1B. Comorbidities | |
|  |  | - 1C. Sociocultural factors | |
|  |  | - The illness/condition: other (list) | |
|  | (2) The technology | - 2A. Material properties | |
|  |  | - 2B. Knowledge to use | |
|  |  | - 2C. Knowledge generated | |
|  |  | - 2D. Supply model | |
|  |  | - 2E. Who owns the IP | |
|  |  | - The technology: other (list) | |
|  | (3) The value proposition | - 3A. Supply-side value (to developer) | |
|  |  | - 3B. Demand-side value (to patient) | |
|  |  | - The value proposition: other (list) | |
|  | (4) The adopters | - 4A. Staff (role, identity) | |
|  |  | - 4B. Patient (passive vs. active input) | |
|  |  | - 4C. Carers (available, type of input) | |
|  |  | - The adopters: other (list) | |
|  | (5) The organization(s) | - 5A. Capacity to innovate | |
|  |  | - 5B. Readiness for this technology | |
|  |  | - 5C. Nature of adoption/funding decision | |
|  |  | - 5D. Extent of changes needed to organisational routines | |
|  |  | - 5E. Work needed to implement and evaluate change | |
|  |  | - The organization: other (list) | |
|  | (6) The wider system | - 6A. Political/policy context | |
|  |  | - 6B. Regulatory/legal issues | |
|  |  | - 6C. Professional bodies | |
|  |  | - 6D. Socio-cultural context | |
|  |  | - 6E. Inter-organisational networking | |
|  |  | - The wider system: Other (list) | |
|  | (7) Over time | - 7A. Scope for adaptation over time | |
|  |  | - 7B. Organisational resilience | |
|  |  | - over time: other (list) | |
| Takeaways | Next steps, based on conclusions (e.g., how to deal w identified complexity)? |  |  |
|  | Study conclusion using NASSS: Will the intervention get adopted, scale, spread, and/or sustain (any)? Or if retrospective, did we identify why it did not succeed? |  |  |
|  | Feedback regarding NASSS application (recommendations) |  |  |
